# Comparative genomic analysis of *Clostridium difficile* ribotype 027 strains including the newly sequenced strain NCKUH-21 isolated from a patient in Taiwan

**DOI:** 10.1186/s13099-017-0219-4

**Published:** 2017-11-29

**Authors:** Haruo Suzuki, Masaru Tomita, Pei-Jane Tsai, Wen-Chien Ko, Yuan-Pin Hung, I-Hsiu Huang, Jenn-Wei Chen

**Affiliations:** 1 Institute for Advanced Biosciences, Keio University, Tsuruoka, Yamagata Japan; 2 Faculty of Environment and Information Studies, Keio University, Fujisawa, Kanagawa Japan; 3 Department of Medical Laboratory Science and Biotechnology, National Cheng Kung University, Tainan, Taiwan; 4 Department of Medicine, College of Medicine, National Cheng Kung University , Tainan, Taiwan; 5 Department of Internal Medicine, Tainan Hospital, Ministry of Health & Welfare, Tainan, Taiwan; 6 Department of Internal Medicine, National Cheng Kung University Hospital, Tainan, Taiwan; 7 Department of Microbiology and Immunology, College of Medicine, National Cheng Kung University, 1 University Road, Tainan, 70101 Taiwan

**Keywords:** *Clostridium difficile*, Ribotype 027 strain NCKUH-21, Genome, Phylogeny, Prophage, Horizontal transfer

## Abstract

**Background:**

*Clostridium difficile* is a Gram-positive anaerobe and the leading cause of antibiotic-associated diarrhea worldwide. The emergence of ribotype 027 (RT027) strains is associated with increased incidence of infection and mortality. To further understand the relationship between *C. difficile* NCKUH-21, a RT027 strain isolated from a patient in Taiwan, and other RT027 strains, we performed whole-genome shotgun sequencing on NCKUH-21 and comparative genomic analyses.

**Results:**

The genome size, G+C content, and gene number for the NCKUH-21 strain were determined to be similar to those for other *C. difficile* strains. The core genome phylogeny indicated that the five RT027 strains R20291, CD196, NCKUH-21, BI1, and 2007855 formed a clade. A pathogenicity locus, *tcdR*-*tcdB*-*tcdE*-*orf*-*tcdA*-*tcdC*, was conserved in the genome. A genomic region highly similar to the *Clostridium* phage $\upvarphi$CD38-2 was present in the NCKUH-21 strain but absent in the other RT027 strains and designated as the prophage $\upvarphi$NCKUH-21. The prophage $\upvarphi$NCKUH-21 genes were significantly higher in G+C content than the other genes in the NCKUH-21 genome, indicating that the prophage does not match the base composition of the host genome.

**Conclusions:**

This is the first whole-genome analysis of a RT027 *C. difficile* strain isolated from Taiwan. Due to the high identity with $\upvarphi$CD38-2, the prophage identified in the NCKUH-21 genome has the potential to regulate toxin production. These results provide important information for understanding the pathogenicity of RT027 *C. difficile* in Taiwan.

**Electronic supplementary material:**

The online version of this article (10.1186/s13099-017-0219-4) contains supplementary material, which is available to authorized users.

## Background

*Clostridium difficile* is a Gram-positive, endospore-forming obligate anaerobe and the current leading cause of antibiotic-associated diarrhea (AAD) within hospital settings worldwide [1]. Estimates have revealed that *C. difficile* infections (CDI) are responsible for 15–25% of all AAD cases [2]. Onset of CDI can be engendered by disruption of the hosts’ gut microbiota by broad-spectrum antibiotic treatments. Aging, prolonged stay in health care settings, and proton-pump inhibitor use all contribute to increased risk of CDI [3]. Although *C. difficile* has been characterized for decades, it first gained prominence in 2003 when an outbreak in North America was found to be caused by a strain with toxin hyperproduction capabilities [4]. The rapid spread of *C. difficile* NAP1/BI/027 strain (PCR ribotype 027 or RT027), which is the same strain characterized with different methods has resulted in outbreaks worldwide, although cases in Asia and Latin America were less reported compared with Europe and North America.

According to a previous case report, NCKUH-21 is the strain isolated from the first severe RT027 CDI in Taiwan, and it contains a deletion of 18 base pairs and a truncated mutation (D117A) in *tcdC* [5]. To further understand the relationship between NCKUH-21 and other RT027 strains including historic strains and hypervirulent strains, we determined the genome sequence of the *C. difficile* strain NCKUH-21 (the accession numbers: BDSN01000001–BDSN01000094) and compared it with other sequenced RT027 strains. We assessed the presence of virulence and antibiotic resistance genes for the NCKUH-21 genome. We also compared the genome sequences of the NCKUH-21 strain with its close relatives to investigate the genome synteny, reconstruct the phylogenetic tree, and identify NCKUH-21 strain-specific genes.

## Methods

Genome sequencing, assembly, and annotation for the strain NCKUH-21, as well as comparative genomics of nine *C. difficile* strains (Table 1), were performed as described in Additional file 1: Materials and methods.

**Table 1:** Analysis of the genomic features of *Clostridium* strains

Organism name	Size (bp)	%G+C	CDS	Source
*C. difficile* R20291	4,191,339	28.8	3543	An epidemic strain, UK, 2006
*C. difficile* CD196	4,110,554	28.6	3485	A patient with CDI, France, 1985
*C. difficile* NCKUH-21	4,217,149	28.4	3810	A patient with severe PMC, Taiwan, 2014
*C. difficile* BI1	4,464,700	28.4	4101	A human strain, USA, 1988
*C. difficile* 2007855	4,179,867	28.7	3811	A bovine strain, USA, 2007
*C. difficile* Z31	4,298,263	29.2	4128	A canine NTCD strain, Brazil, 2009
*C. difficile* CD630	4,298,133	29.1	3908	A patient with severe PMC, Switzerland, 1982
*C. difficile* M68	4,308,325	28.9	3870	A human strain, Ireland, 2006
*C. difficile* M120	4,047,729	28.7	3634	A human strain, UK, 2007
*C. mangenotii* LM2	3,023,790	31.6	2808	A reference genome from the rumen microbiome

%G+C= 100 × (G+C)/(A+T+G+C)

*CDS* number of protein-coding DNA sequences, *PMC* pseudomembrane colitis, *NTCD* non-toxigenic *C. difficile*

### Quality assurance

Genomic DNAs were purified from a pure culture of a single bacterial isolate of NCKUH-21. A BLAST search against a nonredundant database revealed no potential contamination of the genomic libraries.

## Results and discussion

### Genomic features

Illumina MiSeq sequencing was performed to determine the genome sequence of the *C. difficile* strain NCKUH-21. The de novo assembly contained 94 contigs of length 4,217,149 bp, with a G+C content of 28.4% with sequencing coverage of 1611×. Genome annotation yielded a total of 3810 protein-coding sequences (CDSs).

Among the *C. difficile* strains analyzed in this paper, the genome size (Mb) ranged from 4.05 to 4.46, G+C content ranged from 28.4 to 29.2%, and CDS number ranged from 3485 to 4128 (Table 1). The general genomic features for the NCKUH-21 strain were thus similar to those of the other *C. difficile* strains.

### Phylogeny

**Fig. 1:**
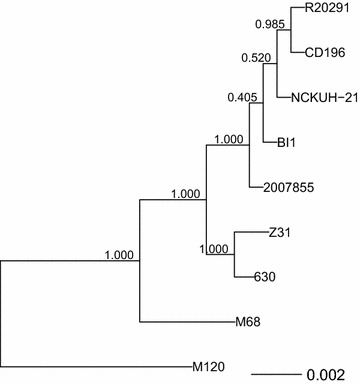
Phylogenetic tree obtained from a concatenated nucleotide sequence alignment of the core genes for the *Clostridium difficile* strains. The horizontal bar at the base of the figure represents 0.002 substitutions per nucleotide site. The FastTree branch support values are indicated

*Clostridium difficile* strains with the same PCR ribotype were reported to cluster together in the phylogenetic trees for the conserved genes [6]. The Roary pipeline produced a total of 8775 homologous groups of genes (“pan-genome”), of which 69 were shared by all the strains used in this study (“core-genome”). The core genome phylogeny indicated that the RT027 strains (R20291, CD196, NCKUH-21, BI1, and 2007855) formed a monophyletic group or clade, joined by the Z31 and 630 strains, followed by the M68 strain, and finally the M120 strain (Fig. 1).

### Synteny

**Fig. 2:**
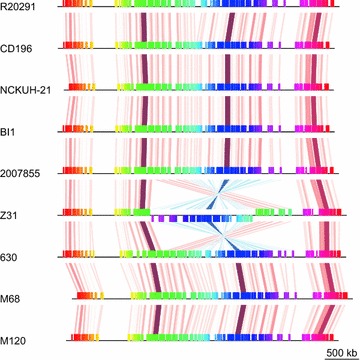
Genome alignment of *Clostridium difficile* strains performed using progressiveMauve (http://darlinglab.org/mauve/user-guide/progressivemauve.html) and visualized using genoPlotR (http://genoplotr.r-forge.r-project.org)

The Mauve Contig Mover (http://darlinglab.org/mauve/user-guide/reordering.html) was used to reorder the contigs of NCKUH-21 relative to the complete genome of *C. difficile* CD196. The genomes of the nine *C. difficile* strains were aligned using progressiveMauve, and this alignment was visualized using genoPlotR to investigate genomic rearrangement (Fig. 2). The genome synteny was determined to be conserved among all but one of the strains. An exception was the Z31 strain with large-scale genomic rearrangement, which had not been previously reported [7].

### Antibiotic resistance and virulence genes

Antibiotic resistance and virulence genes were searched using ABRicate. Homologous DNA sequences for the binary toxin genes *cdtA* and *cdtB* listed in the Virulence Factors Database (accessions of AAF81760 and AAF81761, respectively) were detected in the NCKUH-21 genome [8]. Homologous DNA sequences for the antibiotic resistance genes *cdeA*, *vanRG*, and *vanG* listed in the Comprehensive Antibiotic Resistance Database (accessions of AJ574887.1:371–1697, DQ212986:2259–2967, and DQ212986:5985–7035, respectively) were detected in the NCKUH-21 genome. Although NCKUH-21 showed the genetic potential for becoming resistant to antibiotics, this strain was shown to be susceptible to moxifloxacin (minimum inhibitory concentration 0.5 μg/mL), metronidazole (0.094 μg/mL), and vancomycin (0.5 μg/mL) [5].

The genetic organization of the pathogenicity locus (PaLoc) of the CD630 strain is *tcdR*-*tcdB*-*tcdE*-*orf*-*tcdA*-*tcdC* (locus_tag: CD630_06590, CD630_06600, CD630_06610, CD630_06620, CD630_06630, and CD630_06640) [9]. The gene order was conserved in the NCKUH-21 genome (the accession number: BDSN01000011; locus_tag: NCKUH21_00647, NCKUH21_00648, NCKUH21_00649, NCKUH21_00650, NCKUH21_00651, and NCKUH21_00652). Moreover, another sequence similar to *tcdE* (CD630_06610) was found in the NCKUH-21 genome (locus_tag: NCKUH21_03847) with 83% amino acid identity. The genes *tcdB* and *tcdA* encoding Toxin B and Toxin A (locus_tag: CD630_06600 and CD630_06630; 2366 and 2710 amino acids in length), respectively, of the CD630 PaLoc were determined to be homologous with 48% amino acid identity; additionally, these two genes partly matched a sequence encoding “*N*-acetylmuramoyl-l-alanine amidase LytC” (the accession number: BDSN01000021; locus_tag: NCKUH21_02692; 644 amino acids in length) in the NCKUH-21 genome with 177 and 226 alignment length and 32 and 34% amino acid identity values, respectively. The PaLoc gene homologues may contribute to the virulence and pathogenicity for the *C. difficile* strain NCKUH-21.

### NCKUH-21 strain-specific genes

To identify NCKUH-21 strain-specific genes, we searched the NCKUH-21 strain’s protein homologues in the genome sequences of all *C. difficile* strains by using the gene screen method with TBLASTN in the large-scale blast score ratio (LS-BSR) pipeline. Of the 3810 protein-coding genes identified in NCKUH-21, 3579 were conserved in all the other RT027 strains (R20291, CD196, BI1, and 2007855), and 2832 were conserved in all the *C. difficile* strains used in this study. Among the strains, the largest numbers of NCKUH-21 genes were conserved in the RT027 strains (R20291, CD196, BI1, and 2007855), ranging from 3592 to 3655, followed by other *C. difficile* strains (Z31, 630, M68, and M120), ranging from 3153 to 3431, and finally the outgroup LM2 (761).

A total of 140 protein-coding genes were present in the NCKUH-21 strain but absent in the other strains (Additional file 2: Table S1). The NCKUH-21 strain-specific genes could have been gained on the branch leading to the NCKUH-21 strain, and they could thus be linked to its specific phenotype (e.g., virulence and pathogenicity). Of the 140 NCKUH-21 strain-specific genes, 50 were encoded on the 40,525-bp-long contig sequence of the NCKUH-21 genome (the accession number: BDSN01000034), which showed a 99% identity match to the *Clostridium* phage $\upvarphi$CD38-2 (GenBank accession: HM568888). The genomic region highly similar to the *Clostridium* phage $\upvarphi$CD38-2 was designated as the prophage $\upvarphi$NCKUH-21.

### Prophage $\upvarphi$NCKUH-21

The prophage $\upvarphi$ NCKUH-21 detected in the draft genome for the *C. difficile* strain NCKUH-21 was further confirmed by phage induction examination and electron microscope imaging (data not shown). A previous study suggested that lysogenic $\upvarphi$CD38-2 replicates as a circular plasmid and boosts toxin production in *C. difficile* [10]. The high sequence identity between $\upvarphi$NCKUH-21 and $\upvarphi$CD38-2 suggests that these prophages have a similar role in *C. difficile*.

Reports have revealed that bacterial phages tend to be lower in G+C content than their hosts and that viruses match the G+C content of their hosts [11, 12], including the *C. difficile* bacteriophage $\upvarphi$CD119 [13]. Base composition statistics for the NCKUH-21 genes were calculated as the relative frequency of G+C at third codon positions (GC3). The median GC3 value for the prophage $\upvarphi$NCKUH-21 genes (0.21) was higher than that for the other genes (0.14) in the NCKUH-21 genome. A Wilcoxon rank sum test, which compared the GC3 values between the two groups of genes, was highly significant (P < 2.2e−16). This suggests that the prophage $\upvarphi$NCKUH-21 does not match the base composition of the host genome and may thus have been acquired by horizontal transfer based on the hypothesis of genome amelioration [14].

### Concluding remarks

From 2013 to 2014, three RT027 *C. difficil*e strains were isolated from patients in Taiwan [5, 15, 16]. Among them, NCKUH-21 is the first strain to have a whole-genome sequence for genome comparison. Whether the other two RT027 isolates also carry a complete prophage, what their phylogenetic relation with NCKUH-21 is, and what the relative toxin production level is between the three isolates are all topics for further research.

## Additional files

**Additional file 1.** Materials and methods.

**Additional file 2: Table S1.** Data for *Clostridium difficile* strain NCKUH-21 genes. The columns are as follows: locus_tag, length in amino acids (Laa), G+C content at the third codon positions (GC3), binary number (1 or 0) indicating whether the gene is NCKUH-21 strain-specific (StrainSpecific), gene and product names, and the most similar sequence annotation in the UniRef90 database (FASTA header and organism name).
