# Supplementary material for: Comparative genomic analysis of Clostridium difficile ribotype 027 strains including the newly sequenced strain NCKUH-21 isolated from a patient in Taiwan
Source: Gut Pathog. 2017 Nov 29;9:70. doi: 10.1186/s13099-017-0219-4 (PMC5708112; doi:10.1186/s13099-017-0219-4)
Supplement: Supplementary file 1 — Additional file 1. Materials and methods. [file 13099_2017_219_MOESM1_ESM.docx]

**Additional file 1:**

**Materials and methods**

**Library preparation and sequencing**

*C. difficile* strain NCKUH-21 was grown in BHIS medium overnight, and DNA was extracted using a MasterPure Gram Positive DNA Purification Kit (Epicentre, Madsion,WI, U.S.A.). Whole-genome sequencing was performed using the Illumina MiSeq sequencing platform at the Center for Genomic Medicine, National Cheng Kung University, Taiwan. The sequencer produced 300-bp paired-end reads that were obtained from 550-bp inserts.

**Read quality assessment**

The quality of the reads was checked using FastQC (http://www.bioinformatics.babraham.ac.uk/projects/fastqc/). Prior to assembly, PhiX contaminations were removed from raw reads using bbduk (https://sourceforge.net/projects/bbmap/), and adapter sequences were trimmed using Trimmomatic version 0.36 (http://www.usadellab.org/cms/?page=trimmomatic) [[1](#_ENREF_1)]. The resulting reads were assembled using SPAdes version 3.9.0 (http://cab.spbu.ru/software/spades/) [[2](#_ENREF_2)], and then low-coverage contigs were disregarded. The max sequence length and the N50 length of the contigs were 763,889 and 219,875, respectively.

The contigs of the NCKUH-21 strain were ordered against the complete genome of the closely related strain *C. difficile* CD196 using The Mauve Contig Mover (http://darlinglab.org/mauve/user-guide/reordering.html) [[3](#_ENREF_3)].

**Genome annotation**

Genome annotation was performed using Prokka v1.11, a pipeline comprising several bioinformatic tools [[4](#_ENREF_4)]. Briefly, Infernal+Rfam (http://rfam.xfam.org) found 90 ncRNAs, Aragorn detected 76 tRNA and 1 tmRNA genes, RNAmmer predicted 12 rRNA genes, and Prodigal identified 3,810 protein coding DNA sequences (CDS), of which 176 contained signal peptides identified using SignalP. Of the 3,810 proteins, 1,362 were hypothetical proteins of unknown function, 2,361 were annotated by UniProtKB, and 88 by HAMAP.

We also performed BLASTP similarity searches (with the E value cutoff of 1e-05) of the NCKUH-21 proteins against the UniProt Reference Clusters UniRef90 to assign the most similar (best hit) sequence annotation [[5](#_ENREF_5)]. Of the 3,810 proteins, 3,807 (99.9%) had matched with 3,784 unique records in the UniRef90 database.

ABRicate (https://github.com/tseemann/abricate) was used for screening of genomes for virulence and antibiotic resistance genes with two databases: Virulence Factors Database (VFDB) and The Comprehensive Antibiotic Resistance Database (CARD) [[6](#_ENREF_6), [7](#_ENREF_7)].

**Comparative genomics**

We analyzed nine *C. difficile* strains: five of RT027 strains (R20291, CD196, NCKUH-21, BI1, and 2007855), and other *C. difficile* strains: Z31, 630, M68, and M120 (Table 1). NCKUH-21 was isolated from patients with severe Clostridium difficile Associated Disease in 2014 and 2007 [[8](#_ENREF_8)], respectively. CD196 and BI1 are historic, non-epidemic strains, which were isolated in 1985 and 1988, respectively, and thus the earliest retrospective recorded PCR-ribotype 027 isolates [[9](#_ENREF_9)]. R20291, an epidemic strain isolated in 2006, was used as the representative of hypervirulent strains. The genomic sequences for R20291 and CD196 were generated by Stabler et al. [[9](#_ENREF_9)]. Strain 630 (ribotype 012) is a multidrug-resistant isolate from a patient with pseudomembrane colitis (PMC) in 1982, and was the first *C. difficile* strain for which the complete genome sequence was reported [[10](#_ENREF_10)]. Strain Z31 (ribotype 009) is a non-toxigenic strain, which does not express toxin A and B [[11](#_ENREF_11)]. Strain M120, the non-epidemic strain isolated in 2007 was chosen as the reference strain of ribotype 078 [[12](#_ENREF_12)].

Sequence data were downloaded on 2017-11-02 from the NCBI (ftp://ftp.ncbi.nih.gov/) and PATRIC (ftp://ftp.patricbrc.org/) databases. We retrieved genome sequence data from the NCBI genomes FTP site using the assembly summary report file (the “assembly_summary_refseq.txt” file was used for *Clostridioides mangenotii* LM2 and *C. difficile* strains M120 and M68, while the “assembly_summary_genbank.txt” was used for *C. difficile* strains R20291, CD196, 630, and Z31). The annotated genome sequence for *C. difficile* strains BI1 and 2007855 were downloaded from the PATRIC FTP site.

***Synteny analysis:*** Genome alignment of *C. difficile* strains was performed using the progressiveMauve with default parameters [[13](#_ENREF_13)]. The genome alignment was visualized using genoPlotR [[14](#_ENREF_14)].

***Phylogenetic analysis:*** Homologous groups of protein-coding genes from multiple bacterial genomes were built using Roary [[15](#_ENREF_15)]. Roary was run with the following parameters: "roary -f . -e -n -i 90 -cd 100 data/*gff". Genes present in every genome once (“core-genome”) were aligned using MAFFT [[16](#_ENREF_16)], and concatenated to reconstruct a phylogenetic tree with the GTR+CAT model using the FastTree version 2.1.9[[17](#_ENREF_17)]. *Clostridioides mangenotii* LM2 was used as an outgroup strain to root the tree based on the previous phylogenetic study of 16S rRNA gene sequence data [[18](#_ENREF_18)].

***Gene conservation analysis:*** The conservation of the protein-encoding genes of the strain (NCKUH-21 or CD630) in the genome sequences of all the strains was determined using the gene screen method with TBLASTN in the large-scale blast score ratio (LS-BSR) pipeline [[19](#_ENREF_19)]. The '$prefix_dup_matrix.txt' file (https://github.com/jasonsahl/LS-BSR) was used to determine gene presence and absence for each genome.

***Software:*** Statistical analysis and plots were made in R version 3.3.3 (https://www.r-project.org).

**References**

1. Bolger AM, Lohse M, Usadel B: **Trimmomatic: a flexible trimmer for Illumina sequence data**. *Bioinformatics* 2014, **30**(15):2114-2120.

2. Bankevich A, Nurk S, Antipov D, Gurevich AA, Dvorkin M, Kulikov AS, Lesin VM, Nikolenko SI, Pham S, Prjibelski AD *et al*: **SPAdes: A New Genome Assembly Algorithm and Its Applications to Single-Cell Sequencing**. *J Comput Biol* 2012, **19**(5):455-477.

3. Rissman AI, Mau B, Biehl BS, Darling AE, Glasner JD, Perna NT: **Reordering contigs of draft genomes using the Mauve Aligner**. *Bioinformatics* 2009, **25**(16):2071-2073.

4. Seemann T: **Prokka: rapid prokaryotic genome annotation**. *Bioinformatics* 2014, **30**(14):2068-2069.

5. Suzek BE, Huang HZ, McGarvey P, Mazumder R, Wu CH: **UniRef: comprehensive and non-redundant UniProt reference clusters**. *Bioinformatics* 2007, **23**(10):1282-1288.

6. Chen LH, Zheng DD, Liu B, Yang J, Jin Q: **VFDB 2016: hierarchical and refined dataset for big data analysis-10 years on**. *Nucleic Acids Res* 2016, **44**(D1):D694-D697.

7. Jia B, Raphenya AR, Alcock B, Waglechner N, Guo P, Tsang KK, Lago BA, Dave BM, Pereira S, Sharma AN *et al*: **CARD 2017: expansion and model-centric curation of the comprehensive antibiotic resistance database**. *Nucleic Acids Res* 2017, **45**(D1):D566-D573.

8. Hung YP, Cia CT, Tsai BY, Chen PC, Lin HJ, Liu HC, Lee JC, Wu YH, Tsai PJ, Ko WC: **The first case of severe Clostridium difficile ribotype 027 infection in Taiwan**. *J Infection* 2015, **70**(1):98-101.

9. Stabler RA, He M, Dawson L, Martin M, Valiente E, Corton C, Lawley TD, Sebaihia M, Quail MA, Rose G *et al*: **Comparative genome and phenotypic analysis of Clostridium difficile 027 strains provides insight into the evolution of a hypervirulent bacterium**. *Genome Biol* 2009, **10**(9).

10. Sebaihia M, Wren BW, Mullany P, Fairweather NF, Minton N, Stabler R, Thomson NR, Roberts AP, Cerdeno-Tarrraga AM, Wang HW *et al*: **The multidrug-resistant human pathogen Clostridium difficile has a highly mobile, mosaic genome**. *Nat Genet* 2006, **38**(7):779-786.

11. Pereira FL, Oliveira CA, Silva ROS, Dorella FA, Carvalho AF, Almeida GMF, Leal CAG, Lobato FCF, Figueiredo HCP: **Complete genome sequence of Peptoclostridium difficile strain Z31**. *Gut Pathog* 2016, **8**.

12. He M, Sebaihia M, Lawley TD, Stabler RA, Dawson LF, Martin MJ, Holt KE, Seth-Smith HMB, Quail MA, Rance R *et al*: **Evolutionary dynamics of Clostridium difficile over short and long time scales**. *P Natl Acad Sci USA* 2010, **107**(16):7527-7532.

13. Darling AE, Mau B, Perna NT: **progressiveMauve: Multiple Genome Alignment with Gene Gain, Loss and Rearrangement**. *Plos One* 2010, **5**(6).

14. Guy L, Roat Kultima J, Andersson SGE: **genoPlotR: comparative gene and genome visualization in R**. *Bioinformatics* 2010, **26**(18):2334-2335.

15. Page AJ, Cummins CA, Hunt M, Wong VK, Reuter S, Holden MTG, Fookes M, Falush D, Keane JA, Parkhill J: **Roary: rapid large-scale prokaryote pan genome analysis**. *Bioinformatics* 2015, **31**(22):3691-3693.

16. Katoh K, Misawa K, Kuma K, Miyata T: **MAFFT: a novel method for rapid multiple sequence alignment based on fast Fourier transform**. *Nucleic Acids Res* 2002, **30**(14):3059-3066.

17. Price MN, Dehal PS, Arkin AP: **FastTree 2-Approximately Maximum-Likelihood Trees for Large Alignments**. *Plos One* 2010, **5**(3).

18. Lawson PA, Citron DM, Tyrrell KL, Finegold SM: **Reclassification of Clostridium difficile as Clostridioides difficile (Hall and O'Toole 1935) Prevot 1938**. *Anaerobe* 2016, **40**:95-99.

19. Sahl JW, Caporaso JG, Rasko DA, Keim P: **The large-scale blast score ratio (LS-BSR) pipeline: a method to rapidly compare genetic content between bacterial genomes**. *Peerj* 2014, **2**.

**The FTP paths for *Clostridium* genome sequences downloaded**

| *C. difficile* R20291 | ftp://ftp.ncbi.nlm.nih.gov/genomes/all/GCA/000/027/105/GCA_000027105.1_ASM2710v1/GCA_000027105.1_ASM2710v1_genomic.gbff.gz |
| --- | --- |
| *C. difficile* CD196 | ftp://ftp.ncbi.nlm.nih.gov/genomes/all/GCA/000/085/225/GCA_000085225.1_ASM8522v1/GCA_000085225.1_ASM8522v1_genomic.gbff.gz |
| *C. difficile* NCKUH-21 | ftp://ftp.ncbi.nlm.nih.gov/genomes/all/GCF/002/335/485/GCF_002335485.1_ASM233548v1/GCF_002335485.1_ASM233548v1_genomic.gbff.gz |
| *C. difficile* BI1 | ftp://ftp.patricbrc.org/patric2/current_release/gbf/699034.5.PATRIC.gbf |
| *C. difficile* 2007855 | ftp://ftp.patricbrc.org/patric2/current_release/gbf/699033.6.PATRIC.gbf |
| *C. difficile* Z31 | ftp://ftp.ncbi.nlm.nih.gov/genomes/all/GCA/001/447/175/GCA_001447175.1_ASM144717v1/GCA_001447175.1_ASM144717v1_genomic.gbff.gz |
| *C. difficile* CD630 | ftp://ftp.ncbi.nlm.nih.gov/genomes/all/GCA/000/009/205/GCA_000009205.1_ASM920v1/GCA_000009205.1_ASM920v1_genomic.gbff.gz |
| *C. difficile* M68 | ftp://ftp.ncbi.nlm.nih.gov/genomes/all/GCF/000/210/395/GCF_000210395.1_ASM21039v1/GCF_000210395.1_ASM21039v1_genomic.gbff.gz |
| *C. difficile* M120 | ftp://ftp.ncbi.nlm.nih.gov/genomes/all/GCF/000/210/435/GCF_000210435.1_ASM21043v1/GCF_000210435.1_ASM21043v1_genomic.gbff.gz |
| *C. mangenotii* LM2 | ftp://ftp.ncbi.nlm.nih.gov/genomes/all/GCF/000/687/955/GCF_000687955.1_ASM68795v1/GCF_000687955.1_ASM68795v1_genomic.gbff.gz |
